# Obesity and metabolic dysfunction severely influence prostate cell function: role of insulin and IGF1

**DOI:** 10.1111/jcmm.13109

**Published:** 2017-02-28

**Authors:** Fernando L‐López, André Sarmento‐Cabral, Vicente Herrero‐Aguayo, Manuel D. Gahete, Justo P. Castaño, Raúl M. Luque

**Affiliations:** ^1^ Maimónides Institute of Biomedical Research of Cordoba (IMIBIC) Cordoba Spain; ^2^ Department of Cell Biology, Physiology and Immunology University of Cordoba Cordoba Spain; ^3^ Reina Sofía University Hospital Cordoba Spain; ^4^ CIBER Fisiopatología de la Obesidad y Nutrición (CIBERObn) Cordoba Spain; ^5^ International Campus of Excellence on Agrifood, CeiA3 Cordoba Spain

**Keywords:** insulin, IGF1, prostate, obesity, prostate cancer

## Abstract

Obesity is a major health problem that courses with severe comorbidities and a drastic impairment of homeostasis and function of several organs, including the prostate gland (PG). The endocrine–metabolic regulatory axis comprising growth hormone (GH), insulin and IGF1, which is drastically altered under extreme metabolic conditions such as obesity, also plays relevant roles in the development, modulation and homeostasis of the PG. However, its implication in the pathophysiological interplay between obesity and prostate function is still to be elucidated. To explore this association, we used a high fat–diet obese mouse model, as well as *in vitro* primary cultures of normal‐mouse PG cells and human prostate cancer cell lines. This approach revealed that most of the components of the GH/insulin/IGF1 regulatory axis are present in PGs, where their expression pattern is altered under obesity conditions and after an acute insulin treatment (*e.g. Igfbp3*), which might have some pathophysiological implications. Moreover, our results demonstrate, for the first time, that the PG becomes severely insulin resistant under diet‐induced obesity in mice. Finally, use of *in vitro* approaches served to confirm and expand the conception that insulin and IGF1 play a direct, relevant role in the control of normal and pathological PG cell function. Altogether, these results uncover a fine, germane crosstalk between the endocrine–metabolic status and the development and homeostasis of the PG, wherein key components of the GH, insulin and IGF1 axes could play a relevant pathophysiological role.

## Introduction

The prostate gland (PG) is an exocrine gland tightly influenced by the endocrine milieu, as its development and homeostasis is regulated, for instance, by sexual hormones. Indeed, testosterone, the main sexual hormone responsible for prostate homeostasis, can be locally converted to dihydrotestosterone, which is involved in increasing proliferation and reducing death of PG cells 1, 2, 3. Moreover, a growing body of evidence indicates that the endocrine axis comprising growth hormone (GH), insulin and insulin‐like growth factor 1 (IGF1) is locally expressed 4 in the PG and plays a relevant role in its pathophysiology 5, 6. Thus, earlier studies showed that pituitary‐produced GH is essential for PG development, controlling prostate size 7, 8, and local expression of IGF1 and its receptor (IGF1R), *in vivo* 9, 10 and *in vitro* 11. IGF1 is a well‐known regulator of cell proliferation, differentiation and apoptosis 12, which is mainly produced by the liver 13, but is also expressed locally within the stromal and epithelial cells of PGs, where it can act as an autocrine/paracrine factor 4. Interestingly, studies on mouse models have revealed that IGF1 is essential for the appropriate development of the PG and that GH effects are restricted to the stimulation of IGF1 production 14. Indeed, IGF1 seems to be able to regulate PG at different levels as it has been associated with the development and function of Leydig cells or with the stimulation of GnRH release resulting in LH secretion and expression 15, suggesting an important role on the puberty timing 16; consequently, IGF1 absence induces vestigial prostate development and very low testosterone levels, which results in abnormal perinatal androgenization 17. In addition, GH/IGF1 axis is an important regulatory system for prostatic disorders 4, and its alterations have been linked to prostate cancer development 18. In line with this, insulin has also been shown to be crucial for the correct development of the PG, inasmuch as lack of insulin in non‐obese diabetic mice affects the morphology and function of PGs promoting atrophy of secretory epithelial cells and stroma hypertrophy, leading to development of intraepithelial neoplasia, inflammation and alteration of the secretory process 19. Furthermore, replacement of insulin in this type‐1 diabetic mouse model increased prostate volume, proliferation and apoptosis rate 20, revealing an important role of insulin for prostate growth and homeostasis. Indeed, hyperinsulinemia sensitizes PG to the growth‐promoting effects of testosterone 21 and insulin is also important to maintain the β‐adrenergic and muscarinic cholinergic signalling in prostate, as treatment with this hormone reestablished the expression these receptors in diabetic rats 22.

PG pathophysiology is markedly influenced by obesity 23, 24, 25, a multifactorial chronic disease that represents one of the most serious threats for the global population 26. Obesity is characterized by the deregulation of multiple endocrine–metabolic systems, which are, at least in part, responsible for the well‐known obesity‐related metabolic and cardiovascular complications 27 and, even, with the increased obesity‐associated cancer risk 28. Interestingly, obesity courses with the dysfunction of key regulatory systems, including GH, insulin and IGF1 axes, which are crucial for the correct development and maintenance of several organs 29, including the PG. Nonetheless, although obesity‐associated alterations in GH 29, insulin 30, 31 and IGF1 32 levels, together with the concomitant insulin resistance 33, have been shown to impact the function of several metabolic organs, are thought to be involved in the increased obesity‐associated cancer risk 34, 35, 36 and can negatively affect testosterone levels 37, 38, 39, the actual influence of obesity and the concurrent changes in insulin and IGF1 levels in the physiology of normal and tumoral prostate is still to be elucidated.

Consequently, similar to that previously demonstrated in other endocrine tissues, such as pituitary 31 and mammary gland 40, it has been suggested that the development of obesity could be associated with a certain degree of insulin resistance in the PG and could influence its pathophysiology, modulating the normal expression of several endocrine–metabolic axis, including GH/IGF1/insulin system components, which play a relevant role under normal and pathological conditions. For this reason, we have used here a combination of molecular, cellular and whole‐animal approaches to study the influence of diet‐induced obesity on PG insulin management, and on the gene expression profile of the components of the GH/IGF1/insulin systems at the PG level, as well as the direct role of these hormones (insulin and IGF‐I) in normal and tumoral prostate cells.

## Materials and methods

### Animal models

All experimental procedures were carried out in accordance with applicable guidelines and regulations and following the European Regulations for Animal Care under the approval of the University of Cordoba and the Regional Government Research Ethics Committees. C57BL/6J male mice were obtained at weaning from Janvier‐Labs (Le Genest‐Saint‐Isle, France) and used to: (*i*) establish a model of diet‐induced obesity and (*ii*) isolate and culture normal prostate cells. Animals were housed in sterile filter‐capped cages maintained under standard conditions of light (12‐hrs light/dark cycle; lights on at 07:00 a.m.) and temperature (22–24°C), with free access to sterilized diet and water. To obtain normal primary prostate cells, 8–12‐wk‐old C57BL/6J males fed a standard‐chow diet were used. To generate the diet‐induced obese model, *n* = 10 mice per group were housed individually and fed, starting at 4 weeks until 23 weeks of age, a low‐fat (LF) or a high‐fat (HF) diet [Research Diets Inc, New Brunswick, NJ, USA; LF (10% Kcal fat, 70% Kcal carbohydrates, 20% Kcal proteins), HF (60% Kcal fat, 20% Kcal carbohydrates, 20% Kcal proteins); LF and HF were micronutrients matched diets]. Body weights (BW) were measured once a week. Mice were handled daily to acclimatize them to the experimental procedures and personnel 1–2 weeks before blood sampling or killing. At 23 weeks of age, mice were ip injected with vehicle (control group) or insulin (10 U/kg) and 8 min. later and killed by decapitation without anaesthesia (between 08:00–10:00 hrs). Trunk blood was collected, mixed with EDTA and Miniprotease inhibitor (Roche, Barcelona, Spain) and kept in ice until further centrifugation to obtain plasma. Tissues were immediately excised, weighted and snap‐frozen in liquid nitrogen. Plasma/tissues were stored at −80C until posterior analysis. Mice were weighted before killing, and glucose was measured just afterwards.

### *In vivo* evaluation of metabolic status

As previously reported 41, glucose tolerance tests (GTT; 1 mg/g glucose, ip) were carried out after overnight fasting two weeks before sacrifice, and insulin tolerance tests (ITT; 1 mU/g Novolin, ip) were performed under *ad libitum* fed conditions 1 week before killing (in both cases, beginning between 08:00 and 09:00 a.m.). Ten mice/group (HFD and LFD) were used for this evaluation.

### Determination of whole body composition

Whole body composition (fat and lean mass percentage) was assessed using a Body Composition Analyser E26‐240‐RMT (EchoMRI LLC, Houston, TX, USA) the day before killing (22 weeks of age), as previously reported 42. Ten mice/group (HFD and LFD) were used for this evaluation.

### Assessment of circulating hormones and metabolites

Blood glucose was assessed by glucometer (Accu‐Chek system; Roche Diagnostics, Barcelona, Spain). Gh (EZRMGH‐45K, sensitivity 0.07 ng/ml; Millipore, Billerica, MA, USA), insulin (EZRMI‐13K, sensitivity 0.2 ng/ml; Millipore), Igf1 (AC‐18F1, Immunodiagnostic Systems, sensitivity 63 ng/ml; Fountain Hills, AZ, USA), leptin (EZML‐82K, sensitivity 0.05 ng/ml; Millipore) and corticosterone (AC‐14F1, Sensitivity 0.55 ng/ml Immunodiagnostic Systems, Boldon, UK) levels were assessed using ELISA kits. Ten mice/group (for insulin determination) or 4–5 mice/group (for leptin, Gh, Igf1 and corticosterone determinations) were used. All details regarding the protocol, specificity, detectability and reproducibility for each assay can be accessed at the websites of the indicated companies.

### Normal primary prostate cell cultures from mice

PGs (*n* = 2–3 pooled PGs/experiment, four separate experiments, 3–4 wells/treatment) were dispersed into single cells [following an adapted protocol from 43, 44] and plated at 125.000 cells/well in DMEM with 4.5 g/l glucose and D‐Valine (SeraLab, West Sussex, UK), supplemented with 10% foetal bovine serum (FBS), 1% antibiotic‐antimycotic, and 2 mM L‐glutamine (Sigma‐Aldrich, St. Louis, MO, USA) at 37°C and 5% CO_2_. After 24 hrs, primary prostate cells were pre‐incubated in serum‐free medium for 2 hrs, and subsequently, the medium was replaced with serum‐free medium containing medium alone (control), Igf1 or insulin [Sigma‐Aldrich, 10 nM, doses selected according to previous studies implemented by our group 45, 46, 47, 48]. Culture cells were incubated for 24 hrs, and then total RNA was extracted.

### Prostate cell lines

Two widely accepted human PCa‐derived cell lines, one androgen independent (PC3; ATCC^®^ CRL‐1435™) and one androgen sensitive (LNCaP; ATCC^®^ CRL1740™) were used. PC3 cells were cultured in RPMI‐1640 (Lonza, Basel, Switzerland) complemented with 10% FBS, 1% antibiotic‐antimycotic and 2 mM L‐glutamine. LNCaP cells were cultured in RPMI‐1640 supplemented with glucose (final concentration of 4.5 g/l) and complemented with 10% FBS, 1% antibiotic‐antimycotic and 2 mM L‐glutamine. Cell lines were validated by short tandem repeat analysis (STR; GenePrint^®^ 10 System, Promega, Barcelona, Spain) and checked for mycoplasma contamination by PCR as previously reported 49.

### Proliferation assays

Cell proliferation was determined by Alamar‐Blue colorimetric assay (Invitrogen, CA, USA) as previously reported 50. Briefly, 3.000–5.000 cells/well (3–5 individual experiments, 4 wells/treatment) were seeded in 96‐well plates. Then, cells were 24 hrs‐starved (serum‐free‐medium) and proliferation rate measured, every 24 hrs, for the following 4 days. On the day of measurement, cells were incubated for 3 hrs in 10% Alamar‐Blue/RPMI without FBS and then Alamar‐Blue reduction was measured in FlexStation III (Molecular Devices, Madrid, Spain), exciting at 560 nm and reading at 590 nm. Medium with Alamar‐Blue was replaced with fresh medium with treatments (insulin or IGF1) immediately after each measurement. Results are expressed as percentage *versus* control (cell without treatment).

### Migration capacity assay

The ability of PC3 cells to migrate was evaluated by wound‐healing technique as previously reported 51. Briefly, cells were plated at sub‐confluence in 12‐well plates (four individual experiments, two wells/treatment). Confluent cells were serum‐starved for 24 hrs, and then a wound was made using a 100‐μl sterile pipette tip. Cells were rinsed in PBS and incubated for 16 hrs in medium without FBS in the presence of insulin or IGF1 or medium alone (control group). Migration was calculated by the difference between the wound area before and 16 hrs after the treatment using ImageJ (RSB, Bethesda, MD, USA). Three experiments were performed in independent days, in which 3–4 random pictures along the wound were acquired.

### RNA extraction, reverse transcription and quantitative real‐time PCR (qPCR)

Details of RNA extraction, quantification and reverse transcription have been previously reported elsewhere 52, 53. Specifically, total RNA from fresh pituitary and PG tissues (5 mice/group: LFD and HFD, vehicle or insulin treated) was isolated using Absolutely RNA Miniprep Kit (Agilent, CA, USA), and RNA from primary prostate cell cultures and human cell lines with TRI Reagent (Sigma‐Aldrich), both followed by DNase treatment. Total RNA concentration and purity were assessed using Nanodrop‐2000 spectrophotometer (Thermo Scientific, Waltham, MA, USA). Total RNA (1–2 μg) from each sample was reverse‐transcribed using random hexamer primers and the cDNA First Strand Synthesis kit (MRI Fermentas, Hanover, MD, USA).

The development, validation and application of qPCR to measure the expression levels of different mouse transcripts have been previously reported 31. Briefly, qPCR reactions were performed using the Brilliant III SYBR Green Master Mix and the qPCR Stratagene Mx3000p instrument (Agilent, Santa Clara, CA, USA). Absolute gene expression levels (copy number) were calculated using a standard curve. A No‐RT sample was used as a negative control. For each qPCR reaction, 10 μl of master mix, 0.3 μl of each primer (10 μM stock), 8.4 μl of distilled H_2_O and 1 μl of cDNA (100 ng) were mixed with a program consisting of the following steps: (*i*) 95°C for 3 min., (*ii*) 40 cycles of denaturing (95°C for 20 sec.) and annealing/extension (61°C for 20 sec.) and (*iii*) graded temperature‐dependent dissociation step (55°C to 95°C, increasing 0.5°C/30 sec.). To control for variations in the amount of RNA used and the efficiency of the RT reaction, the expression level of each transcript was adjusted by a Normalization Factor (NF) in each sample obtained from the expression levels of three housekeeping genes (β‐actin, and cyclophilin for mice samples, and β‐actin, GAPDH and HPRT for human cell line samples) using the Genorm program. Expression level of these housekeeping genes did not differ between experimental groups in the tissues analysed (data not shown). Specific primers (Table S1) for the mouse transcripts used were designed with the Primer3‐software.

### Western blot

Tissues (liver and PG, 5 mice/group) were homogenized in RIPA Buffer. Protein concentrations were assessed using Bio‐Rad Protein Assay (Bio‐Rad, Hercules, CA, USA). Samples (20 μg) were mixed with 2× Laemmli buffer, boiled and separated on 10% acrylamide gels, and electrophoretically transferred to Hybond‐ECL nitrocellulose membranes (Amersham Biosciences, Piscataway, NJ, USA). Blots were blocked in 5% nonfat‐dry milk (w/v) dissolved in Tris‐buffered saline containing 0.1% Tween‐20 (TBS‐T) and incubated overnight (4°C) with primary antibodies (1:1000) against phospho‐Ser473 Akt (CS92715; Cell Signaling, Beverly, MA, USA) or total Akt (CS9272; Cell Signaling) in TBS‐T, 5% nonfat‐dry milk. Then, blots were incubated with HRP‐conjugated, goat‐anti rabbit IgG (1:2000; CS70745; Cell Signaling) in 5% dry milk, TBS‐T for 1 hr, washed and exposed (5 min.) to Clarity Western‐ECL Blotting‐Substrate (1705060; Bio‐Rad). Films were scanned using Bio‐Rad Gel DocTM EQ system and images were analysed using ImageJ.

### Statistical analysis

Samples from all groups were processed at the same time. All values are expressed as mean ± S.E.M. or compared with the corresponding controls (set at 100%). All data were tested for normal distribution prior to further analysis. When two groups were compared, *t*‐tests were used to assess significant differences. Multiple groups (BW gaining curves) were compared using a two‐way anova followed by post hoc analysis (Tukey). Correlations between BW and phospho‐AKT levels were assessed by Pearson's correlation test *P* < 0.05 and were considered significant. All statistical analyses were performed using the GraphPad Prism 5.0 software (GraphPad Software Inc., La Jolla, CA, USA).

## Results

**Figure 1:**
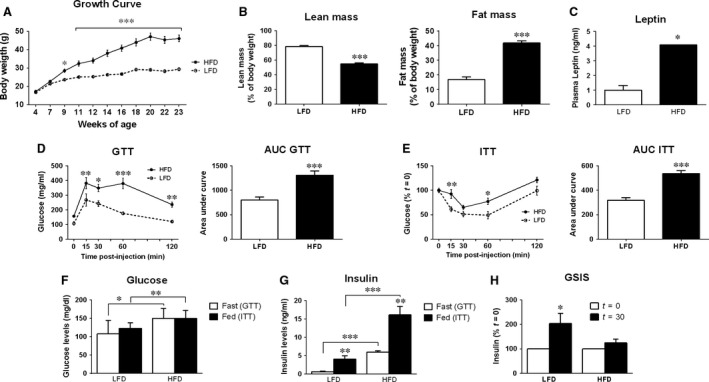
Characterization of the high fat diet–induced obese model. C57BL/6J male mice were fed a low‐fat (LF) or a high‐fat (HF) diet starting at 4 weeks and until 23 weeks of age. Body weight evolution was recorded weekly (**A**) and body compositions by MRI analysis to determine percentage of fat mass and lean mass was performed at 22 weeks of age (**B**). Leptin levels were determined at killing in both groups (**C**). Glucose tolerance test (GTT) was performed at 17 weeks of age. Left graph represent blood glucose levels over time after glucose injection (1 mg/g) and right graph indicate the area under curve (AUC) of the obtained values (**D**). Insulin tolerance test (ITT) was performed at 18 weeks of age. Left graph represents blood glucose levels over time after insulin injection (1 mU/g), and right graph indicates the area under curve (AUC) of the obtained values (**E**). Glucose (**F**) and insulin (**G**) levels were measured at *t* = 0 of ITT and GTT representing fed and fast values, respectively. Glucose‐stimulated insulin secretion (GSIS) was estimated from *t* = 0 and *t* = 30 of the GTT test. *t* = 0 and *t* = 30 represent the time of blood collection, 0 or 30 min. after starting the test (**H**). Data represent mean ± S.E.M. of 10 mice/group. Asterisks (*P < 0.05; **P < 0.01; ***P < 0.001) indicate values that significantly differ from the gender‐matched control group.

As illustrated in Figure 1A, after 5 weeks of feeding, HFD‐fed mice already exhibited a significantly higher BW, which remained elevated until killing. Obese mice also presented significantly lower lean mass and higher body fat mass, by NMR, compared to controls (Fig. 1B). This was also consistent with the observed increase in all fat depots weight measured (*i.e*. visceral, subcutaneous, retroperitoneal, mesenteric and brown fat), liver weight (Table S2), and higher circulating leptin levels (Fig. 1C). Consequently, mice fed a HFD presented a worse GTT (Fig. 1D; left panel) and ITT (Fig. 1E; left panel) profile compared to the LFD controls, as glucose levels were higher in the GTT in response to glucose, and insulin was more inefficient to reduce glucose levels during the ITT in the HFD group. These results were further supported by the areas under curves (AUC) in both tests (Fig. 1D/E; right panels). Accordingly, the model generated displayed hyperglycaemia and hyperinsulinemia, as basal glucose and insulin levels under fast and fed conditions were significantly increased in blood of HFD mice compared with LFD mice (Fig. 1F and G, respectively). Furthermore, in support to the GTT and ITT data, insulin levels were increased 30 min. after glucose injection in LFD mice, but not in HFD mice, suggesting that pancreas of HFD‐fed mice is not appropriately responding to the high glucose levels during the GTT (Fig. 1H). Finally, obese mice presented higher IGF1 and corticosterone levels, whereas GH levels tended to be lower in HFD fed animals (Table S2).

### The PG become insulin resistant under obesity conditions

**Figure 2:**
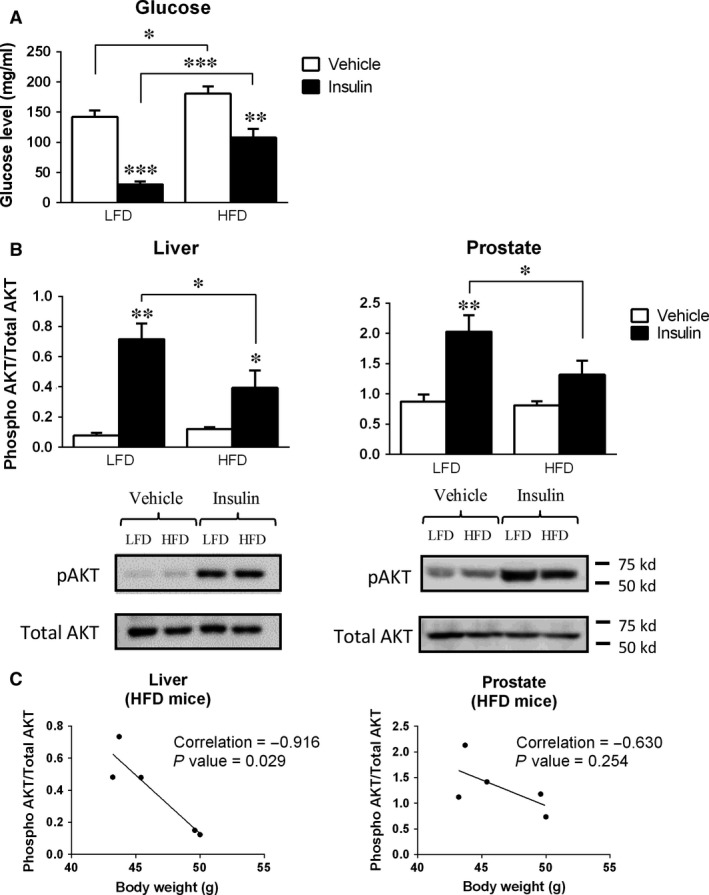
Effect of acute insulin injection on insulin signalling. After 19 weeks of LFD‐ or HFD feeding, all mice (*n* = 5/group) were ip injected with vehicle (control group) or insulin (10 U/kg) and 8 min. later, mice were killed by decapitation. Glucose levels at killing after acute insulin injection were determined (**A**). The ratio of AKT phosphorylation to total AKT in the liver and prostate after insulin injection was determined by western blot (**B**). Correlation between mouse body weight and derangement in insulin signalling (represented as phosphorylation levels of Akt in response to insulin injection) in the liver and prostate of HFD‐fed mice analysed by Pearson's test (**C**). Data represent mean ± S.E.M. of (*n* = 5 mice/group). Asterisks (*P < 0.05; **P < 0.01; ***P < 0.001) indicate values that differ significantly from their respective vehicle‐treated control values.

As expected, glucose levels were significantly lower in mice fed a LFD and HFD injected with insulin (Fig. 2A). However, the percentage of reduction in HFD mice was smaller than in LFD animals (40% of reduction in HFD‐insulin treated compared to HFD‐vehicle *versus* 80% of reduction in LFD‐insulin treated compared to LFD‐vehicle), which supports the state of insulin resistance of the HFD mice. Moreover, HFD mice presented higher basal glucose levels under vehicle‐ and insulin‐treated conditions than the corresponding LFD group (Fig. 2A). Then, to determine whether the PG is an organ sensitive to insulin actions as is the case of the liver, adipose tissue or muscle 31, we analysed the levels of AKT phosphorylation in PGs and livers (used as reference‐control) in vehicle‐ and insulin‐treated mice under LFD and HFD conditions (Fig. 2B). This showed that, although AKT phosphorylation was significantly increased in mice fed with LFD and HFD in response to insulin treatment compared with their respective vehicle‐treated controls, livers of obese mice were resistant to insulin action, for their increment in phospho‐AKT levels was significantly lower than that observed in LFD mice (9.2‐fold induction on insulin‐treated LFD compared to vehicle‐treated LFD mice *versus* 3.2‐fold induction on insulin‐treated HFD compared to vehicle‐treated HFD mice). Remarkably, we found that the PG is also an organ that becomes resistant to insulin actions under an obesity condition and that this state is even more pronounced in the PG than in the liver (Fig. 2B). Specifically, acute insulin injection clearly increased phospho‐AKT levels in lean mice, but not in obese mice at the PG level (*i.e*. the modest increment in AKT phosphorylation induced by insulin in HFD mice was not significant, at variance to that observed in the liver). Interestingly, we found a significant negative correlation between the BW of HFD‐fed mice and the response to insulin injection (activation of AKT signalling) in the liver (*R* = −0.916, *P* = 0.029), whereas this effect was not as obvious in PGs (*R* = −0.630, *P* = 0.254) (Fig. 2C) which might suggest the existence of a tissue‐dependent association between BW and insulin resistance under HFD conditions. Interestingly, this association between mouse BW and derangement in insulin signalling in hepatic tissues of HFD‐fed mice was not observed in LFD‐fed mice (data not shown).

### Expression of components of the GH, insulin and IGF1 axes is regulated in the prostate under obesity conditions and in response to acute insulin treatment

**Figure 3:**
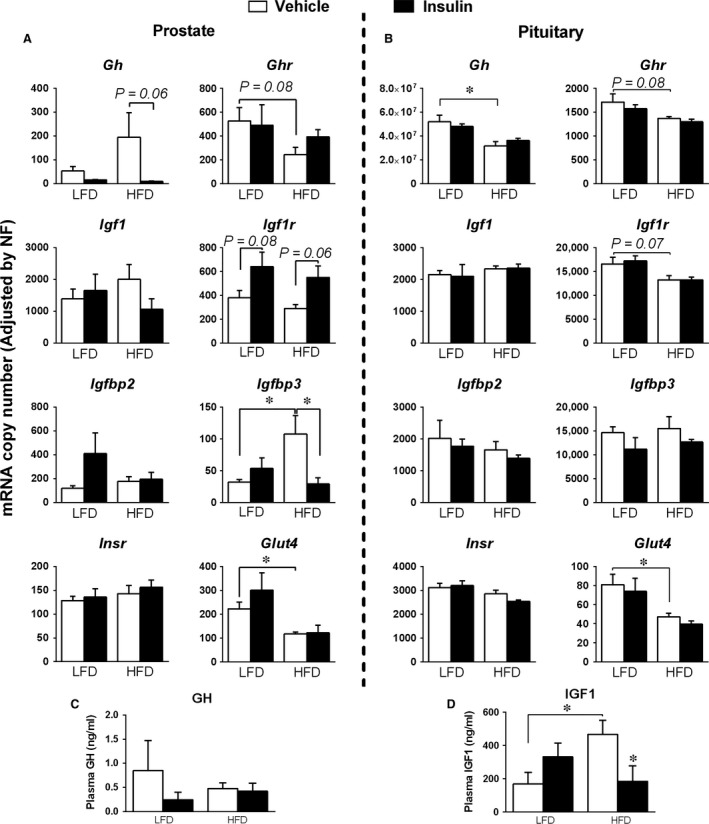
Effects of HFD and acute insulin injection on the expression profile of the prostate glands. mRNA expression level of *Gh*,* Ghr*,* Igf1*,* Igf1r*,* Igfbp2*,* Igfbp3*,* Insr* and *Glut4* was determined by qPCR in the prostate (left panels) and pituitary (right panels) glands of LFD‐ and HFD‐fed male mice injected with vehicle (white columns) or insulin (black columns) for 8 min. Values represent absolute copy number adjusted by a normalization factor (NF; calculated from the expression levels of HPRT, cyclophilin and β‐actin) (**A**). Circulating levels of GH and IGF1 of the four groups were determined at killing (**B**). Values represent mean ± S.E.M. (*n* = 4–5 mice/group). Asterisks indicate values that are significantly differ (*P < 0.05).

Absolute mRNA copy number of several components of the GH, insulin and IGF1 axes, such as *Gh*,* Ghr Insr*,* Igf1*,* Igf1r*, Igf1 binding proteins 2 and 3 (*Igfbp2* and *Igfbp3*), and also of the glucose transporter‐4 (*Glut4*), was determined by qPCR in the PG of male mice fed a LFD (control group; Table 1A). This revealed that the components of the GH/IGF1/insulin axes with lower level of expression were *Igfbp3* and *Gh* (*i.e*. less than 100 copies), followed by *Igfbp2*,* Insr* and *Glut4*, whereas *Ghr*,* Igf1r* and *Igf1* were expressed at high levels (*Igf1 *>* *>*Ghr*>*Igf1r*> Glut4 > Insr>Igfbp2 > Gh>Igfbp3). Interestingly, obesity did not alter drastically the expression levels of some of these components except for an increase in *Igfbp3,* a decrease in *Glut4*, and a non‐significant reduction in *Ghr* (*P* = 0.08) levels in the PGs of HFD‐fed mice compared to LFD‐fed mice (vehicle‐treated; Fig. 3A, white columns). Of note, acute insulin injection tended to increase the expression of *Igf1r* in the prostate of LFD (*P* = 0.08) and HFD (*P* = 0.06) mice as well as to decrease the expression of *Gh* (*P* = 0.06) and *Igfbp3* (*P* < 0.05) only in the prostate of obese (HFD conditions) mice (Fig. 3A, black *versus* white columns).

**Table 1:** Absolute mRNA copy number levels (adjusted by a normalization factor derived from the expression of three housekeeping genes) of the components of the GH, insulin and IGF1 axes on the pituitary and prostate glands of male mice fed a LFD. Data represent means number of copies ± S.E.M. (*n* = 5)

	**A) Prostate**	B) Pituitary
	LFD *(n = 5)*	LFD *(n = 5)*
*Gh*	108.7 ± 57.38	5.2 × 10^7^ ± 5.5 × 10^6^
*Ghr*	526.4 ± 112.1	1712 ± 169.8
*Igf1*	1387 ± 313.1	2154 ± 129.4
*Igf1r*	379.8 ± 61.9	16600 ± 1391.8
*Igfbp2*	118.1 ± 23.4	2016 ± 573.8
*Igfbp3*	31.7 ± 4.4	14620 ± 1221.2
*Insr*	128.4 ± 8.9	3116 ± 179.4
*Glut4*	222.4 ± 27.9	80.9 ± 10.9

To determine whether the changes observed at the PG level under obesity conditions and in response to an acute insulin treatment were tissue specific, we performed the same analysis in the pituitaries of these mice, a tissue where the components of the GH, insulin and IGF1 axes are expressed 31, 54. All the components of these regulatory axes were highly expressed at the pituitary level of adult male mice fed a LFD (control group; Table 1B; *Gh*>>>*Igf1r≥Igfbp3 *>* *>*Insr*>*Igf1 *≥* Igfbp2 *≥* Ghr*>>*Glut4*), and these levels were always higher than those observed at the PG level, except for *Glut4* that presented lower expression levels (Table 1A/B). As expected 31, *Gh* levels were significantly reduced in the pituitary of obese mice (Fig. 3B; white columns). Moreover, the results of mice fed a HFD *versus* LFD at the pituitary level revealed a non‐significant reduction in *Ghr* (*P* = 0.08; similar result in PGs) and *Igf1r* (*P* = 0.07), as well as a significant decrease in *Glut4* expression levels (similar in PGs) (Fig. 3B; white columns). Interestingly, and in contrast to that found at the PG level, acute insulin injection did not induce any specific regulation in the pituitary expression of any component of the Gh, insulin and Igf1 axes analysed of mice fed a LFD or a HFD (Fig. 3B).

To complement these results, plasmatic levels of Gh and Igf1 were determined in these groups of mice. Specifically, no differences in circulating Gh levels were found (Fig. 3C). In contrast, circulating Igf1 levels were significantly increased in obese *versus* lean vehicle‐treated animals (Fig. 3D; white columns), whereas after an acute insulin injection, Igf1 levels only decreased significantly in insulin‐treated HFD mice compared with vehicle‐treated HFD mice (Fig. 3D; black *versus* white columns).

### Direct effects of insulin and IGF1 in normal and tumoral prostate cells

**Figure 4:**
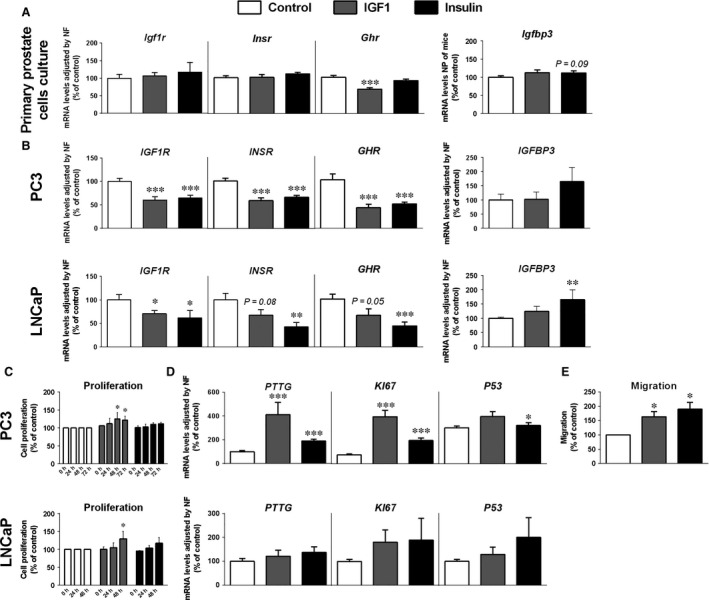
Direct effect of IGF1 and Ins treatment on primary mouse prostate cell cultures and normal‐ and tumoral‐human cell lines. Effect of IGF1 and insulin on *Igf1r*,* Insr*,* Ghr* and *Igfbp3* mRNA expression of primary mouse prostate cell cultures after 24 hrs of treatment determined by qPCR. Values represent absolute copy number adjusted by a normalization factor (NF; calculated from the expression levels of HPRT, cyclophilin and β‐actin) (**A**). Effect of IGF1 and insulin on *IGF1R*,*INSR, GHR* and *IGFBP3* mRNA expression of human prostate cancer PC3 and LNCaP cells after 24 hrs of treatment determined by qPCR. Values represent absolute copy number adjusted by a normalization factor (NF; calculated from the expression levels of HPRT, cyclophilin and β‐actin) (**B**). Effect of IGF1 and insulin on cell proliferation (24, 48 and 72 hrs) of human prostate cancer PC3 and LNCaP cells (**C**). Effect of IGF1 and insulin on mRNA levels of proliferation markers (*PTTG*,*KI67* and *P53*) in PC3 and LNCaP cells after 24 hrs of treatment. Values represent absolute copy number adjusted by a normalization factor (NF; calculated from the expression levels of HPRT, cyclophilin and β‐actin) (**D**). Effect of IGF1 and insulin on the migration of PC3 cells after 14 hrs of treatment (**E**). Values represent the mean ± S.E.M. (*n* = 3–5 individual experiments, 2–4 wells/experiment). Asterisks indicate values that significantly differ from controls (*P < 0.05; **P < 0.01; ***P < 0.001).

To assess the direct role of insulin and IGF1 in the expression of different components of the GH/insulin/IGF1 axes and their implications in key functional parameters of prostate cells such as cell proliferation, migration and PSA secretion, we used normal‐mouse and tumoral‐human prostate cells as models. Particularly, treatment with Igf1 or insulin (24‐hrs incubation) of mouse normal primary prostate cell cultures did not alter the expression of their receptors (*Igf1r* and *Insr*; Fig. 4A, left panel), whereas Igf1, but not insulin, treatment decreased *Ghr* expression (Fig. 4A). Moreover, insulin treatment tended to increase *Igfbp3* expression (*P* = 0.09) in mouse normal primary prostate cell cultures (Fig. 4A, right panel).

In the case of human prostate cancer cell lines (PC3 and LNCaP), 24‐hrs treatment with IGF1 and insulin reduced (or tended to reduce) the expression of IGF1R, INSR and GHR (Fig. 4B, left panels), whereas only insulin increased the expression levels of Igfbp3 in LNCaP cells (Fig. 4B, right panel). Moreover, IGF1, but not insulin, treatment significantly increased cell proliferation in PC3 and LNCaP cells (Fig. 4C). In the case of PC3, but not LNCaP, cells, this was consistent with the prominent effect of IGF1 *versus* insulin treatment on the mRNA levels of two relevant proliferation markers (Ki67 and PTTG), whereas TP53 mRNA levels were not altered (Fig. 4D). Finally, IGF1 and insulin also increased the migration capacity of PC3 cells (Fig. 4E; 16‐hrs treatment).

## Discussion

Obesity is a major health problem associated with severe comorbidities and with drastic impairment of the functioning of several metabolic organs 27, 29. In this scenario, recent studies have demonstrated that obesity induced by high‐fat diets promotes structural and metabolic changes in the PG 23, 24, 25, altering stromal and matrix ultrastructure, as well as the appropriate signalling and modulation by different regulators 23, 24, 25. Although androgen (mainly testosterone) and oestrogen signalling may represent the primary controllers of PG function under normal and pathological conditions 24, the components of the GH, insulin and IGF1 axes have been also shown to play relevant roles in the development, modulation and homeostasis of the PG 4, 19, 20. However, while these neuroendocrine regulatory systems are drastically altered under extreme metabolic conditions such as obesity 29, wherein they associate with key obesity‐associated comorbidities [*e.g*. insulin resistance 55], their implication and regulation in the PG under obesity conditions have not been fully explored hitherto. Accordingly, the present study was devised to determine: (*i*) whether the PG become insulin resistant under obesity conditions in mice, for this may entail important pathophysiological implications, given the relevant role of insulin in the development and normal function of this gland 19, 20, 22; (*ii*) if the expression pattern of the components of the GH, insulin and IGF1 regulatory axes might be altered in the PG under obesity conditions (as occurs in other endocrine organs 31), and also in response to an acute insulin treatment; and finally, (*iii*) the direct role of insulin and IGF1 in the control of normal and pathological PG cell function using primary prostate cell cultures from mice and cultured human cell lines as experimental models.

To achieve these objectives, we first developed a classic mouse model of diet‐induced obesity by feeding male (C57BL/6J background) mice a HFD, and compared to those fed a LFD starting at 4 weeks of age. This model has been widely used as a mouse model of obesity and insulin resistance, as they easily gain weight and develop diet‐induced diabetes 56, 57. Specifically, we found that the group fed a HFD already presented a significantly higher BW 5 weeks after the start of the diet, and maintained this difference until the day of killing. Of note, the BW gain displayed by the HFD mice was associated with a reduction in the lean mass and an increase in fat body mass and leptin levels, compared to the control group, confirming the obese phenotype. As expected, HFD‐fed mice also presented a worst glucose clearance profile and insulin resistance, as indicated by the higher areas under the curve of the GTT and ITT tests in this group compared to the control (LFD) group. Supporting these results, the glucose and insulin levels in fast and fed condition were both higher in the HFD group than in the LFD group, suggesting that the capacity of the pancreas of HFD‐fed mice to respond to metabolic insults was impaired. Consistent with this idea, our data demonstrate that HFD‐fed mice displayed a clearly impaired glucose‐stimulated insulin secretion (GSIS), as LFD‐fed mice exhibited a clear insulin increment in response to the glucose bolus, whereas, in the HFD mice, this rise was completely blunted. Additionally, to confirm the status of peripheral insulin resistance, mice fed a LFD and HFD were injected with insulin and, 8 min. later 31, organs were harvested to determine the phosphorylation levels of a classic insulin‐responsive signalling pathway (AKT, 58). Analysis of the livers of these mice confirmed the peripheral insulin resistance induced by the HFD feeding. Moreover, we demonstrated, for the first time, that the PG, similarly to that observed in other organs 31, develops a drastic insulin resistance in conditions of obesity, as demonstrated by the severely reduced increments of AKT phosphorylation in response to an acute insulin injection, thus reinforcing the idea of a relevant crosstalk between the alterations in the endocrine–metabolic status (*i.e*. obesity) and the dysregulation of the normal metabolic homeostasis of the PG.

To further explore the consequences of the HFD‐induced obesity at the PG level, we carried out a comprehensive characterization of the expression and regulation of key components of the GH, insulin and IGF1 regulatory axes in mice fed a LFD or a HFD, and compared them with the changes observed in the pituitary, an endocrine gland wherein these regulatory axes also play relevant functional roles 45, 48, 59. This analysis confirmed and expanded previous data indicating that the majority of the components of the GH, insulin and IGF1 regulatory axes were expressed in PGs. Particularly, these results are consistent with previous studies showing that the *Ghr* is expressed in the prostate 60, whereas, to the best of our knowledge, our study represents the first piece of evidence showing that *Gh* is also locally expressed in this gland. In this sense, as previous studies have indicated that the role of circulating GH in the PG is circumscribed to the stimulation of the local production of IGF1 4, these data might also suggest the existence of an autocrine/paracrine GH/GHR loop potentially able to stimulate IGF1 production from the PG. Indeed, our results confirmed previous findings showing the expression of *Igf1*,* Igfr* and *Insr* at the PG 4, 9, 10, and demonstrated the local expression of other components of the IGF1/insulin system such as various Igfbps (*Igfbp2* and *Igfbp3*) as well as of other associated proteins, as is the case of the glucose transporter *Glut4*. Most importantly, as previously observed in other tissues 31, 40, our results indicate that the expression pattern of the components of the GH, insulin and IGF1 regulatory axes can be finely modulated in the PG by environmental factors such as the diet. Particularly, we found that PGs of obese mice presented altered expression levels of *Ghr* and *Glut4*, which were similar to those observed in the pituitary of obese mice, but also prostate‐specific changes, such as an up‐regulation in *Igfbp3* expression, which might have some pathophysiological implications as will be further discussed below. Remarkably, the data presented herein also provide primary evidence that an acute insulin treatment can specifically modulate the expression of specific components of the GH, insulin and IGF1 axes at the PG, but not the pituitary level, and that these changes could be different in lean *versus* obese mice. Namely, whereas *Igfr* expression tended to be higher in insulin‐treated LFD and HFD animals, *Igfbp3* expression was severely decreased only in HFD‐fed mice after the acute insulin injection. Although the pathophysiological implications of the changes mentioned herein are still to be elucidated further, these results support the existence of a tight crosstalk between the endocrine–metabolic milieu and the normal homeostasis of the PG, wherein the GH, insulin and IGF1 regulatory axes could to play a important role.

These latter results might be of particular interest, owing to the fact that it has been demonstrated that GH, IGF1 and insulin axes play a direct role in the control of the pathophysiology of prostate cells and, therefore, any dysregulation in the normal expression pattern of these regulatory systems, as the observed under obesity conditions in this study might have some potential pathological implications. Indeed, there is currently strong evidence supporting the existence of a clear association between alterations in the metabolic and hormonal status (*e.g*. changes in circulating insulin, IGF1 and/or GH levels, obesity, diabetes) and a higher risk of developing, and an increased aggressiveness of, certain types of cancer, including prostate cancer 61, 62, 63. In this sense, we did not find any difference in circulating Gh levels in obese mice, likely because of the intrinsic pulsatility of Gh secretion. In contrast, it is plausible that the elevated levels of circulating Igf1 and insulin found in obese *versus* lean vehicle‐treated mice could have some pathophysiological implications at the PG level. Indeed, we used mouse primary prostate cell cultures to determine whether insulin and/or IGF1 could directly be responsible for the alterations in the expression of the components of the GH, IGF1 and insulin axes observed *in vivo* in obese mice (*i.e*. down‐regulation of *Ghr* and up‐regulation of *Igfbp3*). Interestingly, similar to that found *in vivo*, we found that the expression of *Ghr* (but not *Igf1r* or *Insr*) was reduced in response to Igf1, but not insulin treatment in mouse primary prostate cell cultures. Thus, the reduction in Gh signalling at the PG level found *in vivo* (and also *in vitro* in response to Igf1 treatment) may suggest the existence of a negative feedback in this gland, similar to that previously reported at the pituitary 64, wherein excessive circulating Igf1 levels could be exerting a regulatory negative feedback by downregulating of Gh signalling. Moreover, similar to that found *in vivo*, we also observed that *Igfbp3* expression tended to be increased in response to insulin in mouse primary prostate cell cultures, which might have some important pathophysiological implications, as Igfbp3 has been shown to be overexpressed in tumoral *versus* normal‐benign PGs and is associated with enhanced malignancy features in prostate cells 65, 66, 67. Therefore, although insulin and IGF1 did not exert identical actions, likely because of the dissimilar affinity of both hormones to insulin and IGF1 receptors (forming homo‐ and hetero‐dimers) 68, these data reinforce the contention that insulin and IGF1 are main regulators of PG physiology, and anticipate that the changes observed in obese mice could play a role in the pathogenesis of the PG. Nonetheless, the data presented herein using androgen‐independent and androgen‐sensitive prostate cancer cell lines (PC3 and LNCaP) demonstrate that IGF1 and/or insulin can similarly modulate some parameters related to prostate cancer pathophysiology such as proliferation and expression of relevant receptors (*INSR*,* IGFR* or *GHR*), and therefore, we might speculate that these actions of insulin and IGF1 might be androgen independent. Particularly, both hormones altered the expression of *INSR*,* IGFR* and *GHR* and increased the capacity of PC3 cells to migrate. In addition, IGF1 was also able to promote the proliferation of both LNCaP an PC3 cells, which could be likely associated with the prominent increase in the expression of two proliferation markers (*PTTG* and *Ki67*), at least in the case of PC3 cells. Remarkably, these results are in partial agreement with a previous report showing a role of insulin and IGF1 in the modulation of some of these tumoral parameters 69, in particular a consistent stimulatory effect of IGF1 and insulin on cell proliferation in several prostate cancer cell lines 69. Therefore, these results, together with other studies reporting a clear association of the risk of prostate cancer with IGF1 levels, insulin levels and/or insulin resistance 18, 34, 35, 36, 70, reinforce the importance of these two hormones in the pathophysiology of the PG and suggest their putative utility in the prognosis and/or treatment of prostate cancer. In addition, from a clinical point of view, as metformin, an antidiabetic drug associated with a reduction in PCa risk 71, 72, has been shown to reduce insulin levels and insulin resistance, the results presented herein could invite to suggest that one of the putative mechanisms that might help to explain the beneficial actions of metformin on PCa development and progression might be the reduction in insulin resistance at the PG level.

Altogether, the data presented herein demonstrate, for the first time, that the PG becomes severely insulin resistant under diet‐induced obesity in mice and that most of the components of the GH, insulin and IGF1 regulatory axis are present in the PG, wherein their expression pattern is altered under diet‐induced obesity and after an acute insulin treatment. These data, together with the *in vitro* results confirming and expanding the direct role of insulin and IGF1 in the control of normal and pathological prostate cells, demonstrate a relevant crosstalk between the endocrine–metabolic status and the development and homeostasis of the PG, wherein some components of the GH, insulin and IGF1 axes could play a significant pathophysiological role.

## Conflict of interest

The authors confirm that there are no conflicts of interest.

## Author contribution

F.L‐L., A.S‐C. and R.M.L. conceived and designed the project. F.L‐L., A.S‐C., V.H‐A. and R.M.L. acquired the specimens and/or data. F.L‐L., A.S‐C., V.H‐A and R.M.L performed the analysis and interpretation of data. F.L‐L, A.S‐C., M.D.G. and R.L.H wrote the manuscript. V. H‐A. and J.P.C. revised the manuscript. F.L‐L, A.S.C. and R.M.L. performed the statistical analysis. J.P.C and R.M.L obtained funding. R.M.L supervised the work.

## Supporting information


**Table S1** Specific set of primers used for the amplification of mouse and human transcripts by qPCR.

**Table S2** Characterization of the high‐fat diet‐induced obese model. Data represent means ± S.E.M. (*n* = 4–9). Numbers in parentheses indicate the number of samples analyzed for each parameter. Asterisks indicate values that differ between HFD and LFD (*P < 0.05; ***P<0.001).
