# Supplementary material for: Obesity and metabolic dysfunction severely influence prostate cell function: role of insulin and IGF1
Source: J Cell Mol Med. 2017 Feb 28;21(9):1893–904. doi: 10.1111/jcmm.13109 (PMC5571563; doi:10.1111/jcmm.13109)
Supplement: Supplementary file 1 — Table S1 Specific set of primers used for the amplification of mouse and human transcripts by qPCR. [file JCMM-21-1893-s001.docx]

**[Table S](http://www.ncbi.nlm.nih.gov/pmc/articles/PMC4373840/table/pone.0120955.t001/)**1**:** Specific set of primers used for the amplification of mouse and human transcripts by qPCR

|  | Amplified product | Sense | Antisense | Accession Number | Product lenght |
| --- | --- | --- | --- | --- | --- |
| Mice | *B Actin (Actb)* | 5'-CTGGGACGACATGGAGAAGA-3' | 5'-ACCAGAGGCATACAGGGACA-3' | NM_007393.2 | 205 |
|  | *Hprt* | 5'-CAGTCAACGGGGGACATAAA-3' | 5'-AGAGGTCCTTTTCACCAGCAA-3' | NM_013556 | 183 |
|  | *Cyclophilin A (CypA)* | 5'-TGGTCTTTGGGAAGGTGAAAG-3' | 5'-TGTCCACAGTCGGAAATGGT-3' | NM_008907.1 | 109 |
|  | *Gh* | 5'-CCTCAGCAGGATTTTCACCA-3' | 5'-CTTGAGGATCTGCCCAACAC-3' | NM_008117.3 | 142 |
|  | *Ghr* | 5'-GATTTTACCCCCAGTCCCAGTTC-3' | 5'-GACCCTTCAGTCTTCTCATCCACA-3' | BC075720 | 198 |
|  | *Igf1* | 5'-TCGTCTTCACACCTCTTCTACCT-3' | 5'-ACTCATCCACAATGCCTGTCT-3' | NM_010512.3 | 202 |
|  | *Igf1r* | 5'-TGGAGTGCTGTATGCTTCTGTG-3' | 5'-CTGGTTTCGGGTTCATCCTT-3' | NM_010513 | 180 |
|  | *Igfbp2* | 5'-GCGGGTACCTGTGAAAAGAGA-3' | 5'-ACTGCTACCACCTCCCAACA-3' | NM_008342.3 | 135 |
|  | *Igfbp3* | 5'-GGCAGCCTAAGCACCTACCT-3' | 5'-CAACCTGGCTTTCCACACTC-3' | NM_008343 | 97 |
|  | *Insr* | 5'-TCATGGATGGAGGCTATCTGG-3' | 5'-CCTTGAGCAGGTTGACGATTT-3' | NM_010568 | 129 |
|  | *Glut4* | 5'-TGGGAAGGAAAAGGGCTATG-3' | 5'-TGAGGAACCGTCCAAGAATG-3' | AB008453.1 | 117 |
| Human | *B ACTIN (ACTB)* | 5'-ACTCTTCCAGCCTTCCTTCCT-3' | 5'-CAGTGATCTCCTTCTGCATCCT-3' | NM_001101 | 176 |
|  | *HPRT* | 5'-CTGAGGATTTGGAAAGGGTGT-3' | 5'-TAATCCAGCAGGTCAGCAAAG-3' | BT019350 | 157 |
|  | *Cyclophilin A (CYPA)* | 5'-TGGTCTTTGGGAAGGTGAAAG-3' | 5'-TGTCCACAGTCGGAAATGGT-3' | AF022115.1 | 109 |
|  | *GH* | 5'-GACCTAGAGGAAGGCATCCAAA-3' | 5'-AGCAGCCCGTAGTTCTTGAGTAG-3' | NM_000515 | 141 |
|  | *GHR* | 5'-CAGAGGTTAAAAGGGGAAGCA-3' | 5'-AATATGGGCAGCTTGGTGAG-3' | NM_000163.4 | 143 |
|  | *IGF1* | 5'-CATGTCCTCCTCGCATCTCT-3' | 5'-CATACCCTGTGGGCTTGTTG-3' | NM_000618.4 | 161 |
|  | *IGF1R* | 5'-GAGGAAGTGACGGGGACTAAA-3' | 5'-GTGGTGGTGGAGGTGAAATG-3' | NM_000875.4 | 113 |
|  | *IGFBP3* | 5'-CAAGAAAGGGCATGCTAAAGAC-3' | 5'-GTGTGTCTTCCATTTCTCTACGG-3' | NM_001013398.1 | 137 |
|  | *INSR* | 5'-CTTGGGGTTCATGCTGTTCT-3' | 5'-TTCTGTGATTTGGGGTCGTT-3' | NM_000208.3 | 177 |
|  | *PTTG* | 5'-GGCTGTTAAGACCTGCAATAATC-3' | 5'-TTCAGCCCATCCTTAGCAAC-3' | NM_004219.2 | 101 |
|  | *KI67* | 5'-GACATCCGTATCCAGCTTCCT-3' | 5'-GCCGTACAGGCTCATCAATAAC.-3' | NM_002417 | 139 |
