# Supplementary material for: Obesity and metabolic dysfunction severely influence prostate cell function: role of insulin and IGF1
Source: J Cell Mol Med. 2017 Feb 28;21(9):1893–904. doi: 10.1111/jcmm.13109 (PMC5571563; doi:10.1111/jcmm.13109)
Supplement: Supplementary file 2 — Table S2 Characterization of the high‐fat diet‐induced obese model. Data represent means ± S.E.M. (n = 4–9). Numbers in parentheses indicate the number of samples analyzed for each parameter. Asterisks indicate values that differ between HFD and LFD (*P < 0.05; ***P<0.001). [file JCMM-21-1893-s002.docx]

**[Table](http://www.ncbi.nlm.nih.gov/pmc/articles/PMC4373840/table/pone.0120955.t001/)** S2**. Characterization of the high-fat diet-induced obese model.** Data represent means ± SEM (n=4-9). Numbers in parentheses indicate the number of samples analyzed for each parameter. Asterisks indicate values that differ between HFD and LFD (*, p<0,05; ***, p<0,001)

|  | LFD | HFD |
| --- | --- | --- |
| **Body Weigth (g)** | 29.52 ± 1.03 (5) | 49.2 ± 1.34*** (4) |
| **Visceral fat (g)** | 0.80 ± 0.13 (5) | 2.27 ± 0.27* (4) |
| **Subcutaneous fat (g)** | 0.25 ± 0.05 (5) | 1.72 ± 0.18* (4) |
| **Retroperitoneal fat (g)** | 0.12 ± 0.03 (5) | 0.71 ± 0.05* (4) |
| **Mesenteric fat (g)** | 0.21 ± 0.04 (5) | 1.24 ± 0.08* (4) |
| **Brown fat (g)** | 0.25 ± 0.03 (5) | 0.55 ± 0.04* (4) |
| **Liver weight (g)** | 1.27 ± 0.03 (5) | 1.92 ± 0.15* (4) |
| **IGF1** | 168.44 ± 70.49 (5) | 467.87 ± 84.76 (4) |
| **Costicosterone** | 63.19 ± 3.99 (5) | 104.08 ± 4.15 (4) |
| **GH** | 0.85 ± 0.63 (5) | 0.46 ± 016 (4) |
